# Response to the Letter “Body Mass Index, Height, and Head and Neck Cancer Risk: The Japan Public Health Center-based Prospective Study”

**DOI:** 10.2188/jea.JE20250399

**Published:** 2025-12-05

**Authors:** Norie Sawada, Tomohiro Shinozaki, Seitaro Suzuki, Taiki Yamaji, Motoki Iwasaki, Manami Inoue, Shoichiro Tsugane

**Affiliations:** 1 Division of Cohort Research, National Cancer Center Institute for Cancer Control, Tokyo, Japan; 2 Department of Information and Computer Technology, Faculty of Engineering, Tokyo University of Science, Tokyo, Japan; 3 Division of Epidemiology, National Cancer Center Institute for Cancer Control, Tokyo, Japan; 4 Division of Prevention, National Cancer Center Institute for Cancer Control, Tokyo, Japan; 5 International University of Health and Welfare Graduate School of Public Health, Tokyo, Japan

Dear Editor,

We appreciate the thoughtful comments of Lai et al^1^ regarding our study on the association of body mass index (BMI), height, and risk of head and neck cancer (HNC) in the Japanese population.^2^ The letter points out the importance of reporting effect modification, particularly regarding high BMI groups.

Based on their suggestion, we incorporated product terms between all variables (including BMI) and smoking (never/ever smokers) into multivariable Cox models stratified by smoking status (Table 1). We conducted the same analysis for alcohol consumption categories (non- and occasional/regular drinkers, including heavy drinkers) (Table 2), defining regular drinkers as subjects who drank above once per week and accordingly merging the categories of regular and heavy drinkers in the original paper.^2^

**Table 1.** The association between body mass index and risk of head and neck cancer by smoking status

BMI category, kg/m^2^	Never smokers (*n* = 60,607)			Former and current (ever) smokers (*n* = 40,318)			Modification by smoking			
		
	HR^a^	95% CI^a^		HR^a^	95% CI^a^		ratio of HRs^b^	95% CI		*P*
<18.5	2.65	1.16	6.05	3.09	1.54	6.2	1.17	0.40	3.44	0.78
18.5 to 20.9	0.94	0.49	1.79	2.27	1.47	3.5	2.43	1.11	5.30	0.03
21 to 22.9	1.05	0.62	1.8	1.56	1.02	2.37	1.48	0.75	2.93	0.26
23 to 24.9	1	Ref		1	Ref		1	Ref		
25 to 27.4	1.13	0.65	1.98	1.02	0.61	1.68	0.90	0.42	1.90	0.78
≥27.5	1.66	0.91	3.03	1.04	0.55	2.00	0.63	0.26	1.52	0.30

CI, confidence interval; HR, hazard ratio.

^a^For hazard ratios reported in the original paper.^2^

^b^The ratio of HRs was estimated using stratified Cox models, with smoking or drinking as the stratification variable. The models included product terms between the stratification variable and all other covariates. The reported ratio of HRs represents the exponentiated coefficients of the product terms between body mass index and the stratification variable. Firth’s bias correction was applied in all models following the method described in the original study.^2^

**Table 2.** Association between body mass index and risk of head and neck cancer by drinking status

BMI category, kg/m^2^	Non- and occasional drinkers (*n* = 61,358)			Regular drinkers^b^ (*n* = 39,242)			Modification by drinking			
		
	HR^a^	95% CI^a^		HR	95% CI		ratio of HRs^c^	95% CI		*P*
<18.5	2.25	1.04	4.89	3.23	1.56	6.67	1.44	0.50	4.15	0.50
18.5 to 20.9	0.91	0.50	1.67	2.21	1.41	3.46	2.42	1.14	5.12	0.02
21 to 22.9	0.86	0.50	1.47	1.64	1.08	2.51	1.91	0.96	3.79	0.06
23 to 24.9	1	Ref		1	Ref		1	Ref		
25 to 27.4	1.07	0.62	1.85	1.03	0.62	1.71	0.97	0.46	2.04	0.93
≥27.5	1.07	0.55	2.10	1.54	0.87	2.73	1.44	0.60	3.48	0.42

CI, confidence interval; HR, hazard ratio.

^a^For hazard ratios reported in the original paper.^2^

^b^Regular drinkers were defined as those who drink at least once a week, regardless of the amount consumed (including heavy drinkers).

^c^See the Footnote in Table 1 for analysis of the ratio of HRs.

Regarding effect modification by smoking, the hazard ratio (HR) for the lowest BMI (<18.5 kg/m^2^) was similarly high, regardless of smoking status. However, the 18.5–20.9 kg/m^2^ group was not associated with increased risk of HNC among non-smokers but was associated with increased risk in smokers (ratio of HRs: 2.43; *P* = 0.03). Also, at 21–22.9 kg/m^2^, the point estimate in ever smokers was higher than that in never-smokers, but the *P*-value was not statistically significant (ratio of HRs: 1.48; *P* = 0.26). In contrast, although the point estimates in never-smokers were higher than those in smokers at 25–27.4 kg/m^2^ and ≥27.5 kg/m^2^ compared to 23–24.9 kg/m^2^, the confidence intervals were also wide and the *P*-values were large (*P* = 0.78 and *P* = 0.30, respectively). Accordingly, the difference in HR by smoking status at high BMI may not be statistically well-defined.

Regarding effect modification by drinking status, HR for <18.5 kg/m^2^ was similarly high compared to 23–24.9 kg/m^2^, regardless of alcohol consumption. However, no increase in HR was observed in non-drinkers at 18.5–20.9 kg/m^2^ or 21–22.9 kg/m^2^, whereas an increase was observed in drinkers (ratio of HRs: 2.42; *P* = 0.02 and ratio of HRs: 1.91; *P* = 0.06, respectively). Although the HR for a BMI of 25–27.4 kg/m^2^ showed no association regardless of alcohol consumption, the point estimates in regular drinkers were higher than those for non-drinkers at ≥27.5 kg/m^2^. Again, however, the confidence intervals were wide, and the *P*-values were large (*P* = 0.42). As also seen for smoking status, these results suggest that the difference in HR by drinking status at high BMI may not show statistically significant effect modification.

Previous papers^3^ have reported that lower BMI enhanced smoking- and drinking-related incidence of oral cavity/pharyngeal cancer, and that only lower BMI showed higher risk in both never and ever drinkers,^4^ or in ever drinkers.^5^ In contrast, as Lai et al mentioned, effect modification by smoking or drinking remains unclear, particularly with regard to high BMI. The results in our original paper^2^ showed that the point estimate for HNC risk at high BMI (≥27.5 kg/m^2^) was higher in never smokers than ever smokers, but our additional analyses did not suggest that high BMI was associated with different effects by smoking status. Nevertheless, the number of cases in the ≥27.5 kg/m^2^ groups was relatively small, and power for related interaction tests may accordingly have been limited. Confirmation of our present results will require a large number of cases. This study accordingly also highlights the importance of large-scale studies, such as those afforded by cohort consortia.

## References

[1] Lai SW, Liao KF. Letter to the editor “Body Mass Index and Head and Neck Cancer Risk”. J Epidemiol. 2025;35(12):518. 10.2188/jea.JE2025021840484675

[2] Suzuki S, Yamaji T, Iwasaki M, . Body mass index, height, and head and neck cancer risk: the Japan Public Health Center-based Prospective Study. J Epidemiol. 2025;35(4):170–177. 10.2188/jea.JE20240033 39183034 PMC11882347

[3] Gaudet MM, Kitahara CM, Newton CC, . Anthropometry and head and neck cancer: a pooled analysis of cohort data. Int J Epidemiol. 2015;44:673–681. 10.1093/ije/dyv059 26050257 PMC4481608

[4] Chen Y, Lee YA, Li S, . Body mass index and the risk of head and neck cancer in the Chinese population. Cancer Epidemiol. 2019;60:208–215. 10.1016/j.canep.2019.04.008 31071526

[5] Radoï L, Paget-Bailly S, Cyr D, . Body mass index, body mass change, and risk of oral cavity cancer: results of a large population based case-control study, the ICARE study. Cancer Causes Control. 2013;24:1437–1448. 10.1007/s10552-013-0223-z 23677332

